# Cook Co. Hospital—Dr. J. P. Ross

**Published:** 1876-02

**Authors:** D. A. K. Steele


### COOK COUNTY HOSPITAL.

SERVICE OF PROF. JOS. P. ROSS. (Reported by D. A. K. STEELE, M.D.)

### CASE I. *Peritonitis—Death.*

D. F., æt. 24; Irish, laborer; admitted Aug. 18th, 1875. Has been in poor health for the past year, suffering from "erysipelas of the legs," as he called it. Six weeks ago he began to suffer from headache, anorexia and pain in the sides. About three weeks ago he was attacked with nausea and vomiting at night and with severe diarrhœa. The latter has continued to date, with occasional vomiting after eating. He has been steadily losing flesh in the meantime. Pulse 132, and feeble; temperature, 101° F.; respirations, 36; tongue dry and red at the edges; pain in the head, sides and abdomen; four stools daily, thin and yellow; abdomen very tympanitic. Was ordered emulsion of laudanum and turpentine, with eight grains of quinine daily.

Aug. 20th, A. M.—No better, did not sleep well; four stools in the night, three this morning. Abdomen still distended with gas; tongue furred and dry, red at tip. Pulse, 114, small and feeble; temp., 100.2; resp., 36. P. M.—Pulse, 120; temp., 99.2; resp., 32.

21st, A. M.—Pulse, 90; temp., 98; resp., 30. Rested well; three stools during night; pulse larger and stronger; tongue covered with heavy white fur. Heart is displaced upwards; fluctuations found all over abdomen, which is very tender on pressure. P. M.—Pulse, 120; temp., 101.2; resp., 36. Two stools during the day. Ordered one grain of crude opium every four hours, and turpentine stupes to the abdomen.

22nd, A. M.—Slept fairly; pain not so severe; abdomen distended with fluid, and exquisitely tender on pressure. Pulse a little stronger; tongue coated white; pulse, 90; temp., 98; resp., 36; considerable dyspnœa; two stools last night, one this morning.

23rd.—Pulse, 132; temp., 101; resp., 24; pulse very small and feeble. Has not slept. Three stools during night. Cannot retain his medicine.

27th.—Slept well; does not suffer much pain; vomits everything but milk. Tongue still heavily furred and dry. Pulse, 108.

26th.—Pulse, 126 very weak; vomits frequently.

Sept. 4th.—Patient continued sinking and died to-day.

*Autopsy*, twelve hours after death, revealed extensive inflammation of gastro-enteric tract, also of peritoneal membrane; firm adhesions of all abdominal viscera. Abdomen contains a quantity of flaky serum.

Case II. *Cirrhosis—Pleurisy with Effusion—Endopericarditis—Death.*

C. J., æt. 68; German, laborer; admitted Aug. 28th, 1875. Patient unable to give an explicit history, but states that he has had a cough for three or four months. Three weeks ago he had a chill, followed by fever and an aggravation of the cough. Expectorated thick, yellowish white sputa. He is well nourished, but the circulation is sluggish in hands and face. Abdomen, ascitic. Admits that he has been a hard drinker. Pulse, 120, feeble and irregular; temp., 99.2; resp., 26. Tongue, furred and whitish.

Percussion dullness, purely bronchial breathing, crepitant rales, and increased vocal resonance over lower right chest. Exaggerated respiratory murmur over upper portion of right and whole of left chest. Hands and face cyanosed; dyspnœa excessive. Was ordered, every two hours, quinine and opium.

Sept. 1st.—Patient weaker; anorexia; dyspnœa more marked. Pulse extremely feeble and irregular.

Sept. 8th.—Death.

*Autopsy*, eight hours after death. The right pleural cavity was filled with fluid, right lung collapsed; no crepitation.

Pericardial sac filled with fluid; its inner surface and the outer surface of heart covered with lymph. Left ventricle very much hypertrophied; aortic and mitral valves thickened and roughened. Liver cirrhosed, small and nodulated, spleen softened and disorganized, abdomen filled with fluid.

Kidneys congested and indurated; smaller than normal. Dura mater everywhere adherent to cranium; membranes covered in places by lymph and adherent to brain; venous congestion of the latter; its substance softened.

Case III. *Alcoholism.*

J. C., æt. 39; American, printer; admitted Sept. 10th, 1875. States that he has been drinking quite freely for the past three weeks, and during the last week has been drunk nearly all the time, eating little or nothing. Has had almost continuous diarrhœa during this period; recently the stools have become dysenteric. He is well nourished, skin about normal, eyes congested, pupils dilated. Constant muscular tremor, and marked insomnia. Discharged, cured, after treatment by opium, chloral, and potassic bromide.

CASE IV. *Severe Colica Pictorum and Palsy from use of a Cosmetic.*

Maggie P., æt. 21; American, housewife; admitted July 12th, 1875. Was always well enough to do her work until two weeks ago, when she had severe gastric and intestinal pain with vomiting; her bowels had been loose, but then became constipated. For the past four years she had been using, daily, a cosmetic of carbonate of lead. Previous similar attacks were relieved by treatment.

Patient is anæmic; pulse, 90; temp., 98; tongue furred and dry; blue line around margins of gums; eyes puffy; appetite poor; constant pains across the stomach, and vomiting. Paralysis of the extensor muscles of forearm giving the "drop wrist"; metallic taste in the mouth.

Recovery, Sept. 18, after exhibition of ten grains of potassic iodide three times daily, and disuse of the cosmetic.

CASE V. *Double Pleurisy—Paracentesis—Death.*

T. B., æt. 35; Irish, coachman; admitted Oct. 11th, 1875. Patient was well until one week ago when he exposed himself to a draft of cold air while perspiring freely. The same night he had a severe chill, followed by fever and pains in the head, body and limbs, also severe sticking pains in the right side below the nipple; could not take a long breath, began to cough, pains aggravated by coughing. These symptoms have continued ever since. For the last four days has been unable to lie upon the left side.

Patient is well nourished, skin hot, tongue heavily coated; pulse, 114; temp., 101; resp., 36. Complains of loss of appetite; pain in right chest; he coughs and expectorates a thick, white, tenacious sputum.

Respiration almost wholly abdominal, loss of motion and partial obliteration of intercostal spaces in right side. Percussion gives flatness over the right chest anteriorly and posteriorly below the line of the nipple; no distinct postural change of line of dullness.

Diminished respiratory mumur and absence of vocal resonance over lower wo-thirds of right chest; exaggerated respiratory murmur throughout left chest, with mucous and sonorous rales; a few moist rales are also heard at apex of right chest; tubular respiration below right nipple anteriorly. Taking of a mixture of ammon muriat., tart. emet. and morphia, the patient did pretty well until Oct. 14th. That morning he complained of severe lancinating pains in lower part of left chest, and the examination detected distinct pleuritic friction below the nipple; applied hot flaxseed poultice, and ordered Dover's powders of gr. x each. In the afternoon the condition of the patient grew much worse; dyspnœa and cyanosis of the face had risen to an alarming degree, and on physical examination an exudation was found in the left pleural cavity. Under such circumstances Dr. Ross performed paracentesis of the right thorax, and, by means of the aspirator, withdrew about two quarts of a straw-colored serum. The patient felt great relief immediately after the operation, but after a restless night he rapidly failed, and died on the next morning.

*Autopsy*, six hours after death. Extensive exudation of lymph upon costal and pulmonary pleura of right side; about two quarts of serum in right pleural cavity. Left pleura roughened slightly, old adhesions present; about one quart of serum in left pleural cavity.

